# An Explainable Quality-Aware ECG–PCG Fusion for Cardiovascular Disease Detection Using Robust Feature Modeling

**DOI:** 10.3390/diagnostics16142296

**Published:** 2026-07-22

**Authors:** Faiq A. Mohammed Bargarai, Sagvan Ali Saleh, Abdulkadir Sengur

**Affiliations:** 1 Networks Department, Bardarash Technical Institute, Akre University for Applied Sciences, Kurdistan Region, Akre 42004, Iraq; faiq.ahmed@auas.edu.krd; 2 Electrical and Computer Engineering, College of Engineering, University of Duhok, Duhok 99454, Iraq; sagvan.saleh@uod.ac; 3 Electrical-Electronics Engineering Department, Technology Faculty, Firat University, Elazig 23119, Turkey

**Keywords:** cardiovascular diseases, electrocardiograms, phonocardiograms, quality-aware fusion, explainability

## Abstract

**Background/Objectives:** To develop a fast and interpretable multimodal framework for the automatic detection of cardiac abnormalities using electrocardiogram (ECG) and phonocardiogram (PCG) signals. **Methods:** A multimodal classification scheme was designed by combining ECG and PCG recordings. For each modality, tailored preprocessing and temporal and spectral feature extraction were applied. The resulting information was fused through a quality-aware strategy that prioritized more reliable signal segments. The explainability results showed that the model focused on physiologically meaningful regions, providing supportive interpretability for its predictions. Experiments were conducted on the PhysioNet/CinC 2016 heart sound dataset, including normal and pathological recordings, using 10-fold cross-validation. **Results:** The proposed method achieved a mean F1 score of 97.2%, an accuracy of 95.6%, a specificity of 88.6%, and a sensitivity of 97.7%. In addition, the lightweight preprocessing and fast feature extraction pipeline allowed the full 10-fold cross-validation procedure to be completed in only 66 s. **Conclusions:** The proposed ECG-PCG framework provides a fast, accurate, and interpretable solution for automated cardiac abnormality detection and appears well suited for real-time cardiac screening applications.

## 1. Introduction

CVD is an increasing global health risk, and its timely and accurate diagnosis, as such, is more than ever necessary [1]. The traditional diagnosis depends heavily on single-mode signals and comprises processes that are inconvenient to the patient [2]. Even though invasive ones provide cardiac health with complete information, they are inconvenient in nature and require sophisticated and expensive equipment [3]. Additionally, affordable, low-cost technologies such as auscultation with the use of Artificial Intelligence (AI) are highly dependent on the experience of a clinician and can lead to misdiagnosis in patients with complicated cardiac diseases [4]. Even routine tests such as ECG tests, although easily accessible and not invasive, can be insensitive in detecting certain diseases and lead to delayed treatment and development of the disease [5]. Therefore, clinical practice now more and more relies on the multi-modal detection data, with such signals as PCG, ECG, and cardiac color Doppler echocardiography, for complete diagnosis [6]. As a specific example, co-analysis of the ECG and PCG signals can aid in the determination of important cardiac hemodynamic parameters [7]. Moreover, certain ECG morphological changes, like ST-segment elevation or depression and T-wave inversion or flatness, are established markers of myocardial infarction or ischemia, which in most cases are due to Coronary Artery Disease (CAD).

New developments in cardiac signal processing have investigated several approaches to fusion of PCG and ECG modalities. Kalatehjari et al. [8] introduced a hybrid Convolutional Neural Networks (CNN) and BiLSTM network that learns independent temporal–spectral representations from both ECG and PCG, then fuses stream-wise predictions with a bilinear layer to output the final decision. The network is modality-specific encoders to retain electrical and acoustic cues, applies five-fold cross-validation, and achieves strong generalization with 97.77% accuracy on the PhysioNet CinC 2016 dataset. Depth in networks and bilinear fusion add memory and compute requirements, which can be a bottleneck factor in real-time execution on embedded systems. Chandrasekhar et al. [9] suggested Polynomial Jacobian Matrix-based Deep Jordan Recurrent Neural Network for heart cycle sequence modeling. The preprocessing pipeline is band-limited filtering, HEEMD trend removal and denoising, and RFFC clustering with PDF-SLO feature selection to eliminate redundancy. The classifier gets about 97.33 percent on CinC 2016 using training data as combined ECG and PCG, and fast inference when features are ready. The article is concerned with certain specific cardiac disease like atrial fibrillation and congestive heart failure, and the authors propose taking temporal and morphological descriptors to larger clinical scenarios. Potes et al. [10] used a two-branch ensemble in which a feature-based AdaBoost-abstain classifier uses 124 time–frequency descriptors, and a CNN uses cycle-wise spectrotemporal maps divided over four bands. A voting rule aggregates branch outputs to achieve maximum challenge score of 0.8602 and high referral sensitivity of 0.9424 on the hidden test set. The ensemble is sensitivity-biased, elevating false positives, and, hence, specificity is lower than that achieved by individual base learners, which is acceptable for screening but not optimal for confirmatory diagnosis. Zabihi et al. [11] circumvented explicit segmentation in order to reduce computational expense and segmentation mistakes. They mined multi-domain descriptors directly from full PCG recordings, chose 18 features with a wrapper approach, and learned an ensemble of 20 feedforward neural networks. The method attained a 0.8590 total score for CinC 2016. Since the initial selection emphasized more on high anomaly discrimination compared to quality grading, performance suffered when the organizers weighted signal quality higher, suggesting the requirement of explicit quality modeling in pipeline design. Chowdhury et al. [12] presented a PCG pipeline of denoising, compression, segmentation of cycles, and classification. Cepstral and spectrographic cues’ effectiveness in murmur detection and cycle characterization is reflected in the final classification achieved through extraction of features from Mel-scaled power spectrograms and MFCCs and classification by a five-layer feedforward DNN. The system obtained about 97.10 percent test accuracy on combined sets. The technique is dependent on sufficient labeled data, and segmentation is compromised with pathological murmurs that hide S1 or S2, smearing temporal boundaries. Rubin et al. [13] projected one-dimensional PCG segments into two-dimensional Mel Frequency Cepstral Coefficients (MFCC) heat maps and used a deep CNN to separate normal and abnormal recordings. Segmentation was used only to superimpose 3-second windows at S1, keeping cycle normalization to a minimum. The model achieved 84.8 percent at Phase II of the competition. Whole-recording classification utilized averaging segment probabilities, a null aggregation that could be too sparse for pathological bursts and could be enhanced with sequence or attention models. Tschannen et al. [14] integrated deep structured features and standard summary descriptors. The wavelet-based CNN generates high-level features that retain morphology and timing information, power spectral density, and state statistics being summary statistics of the entire record. A Support Vector Machines (SVM) back end performs final classification with an overall 0.812 on the hidden test set. The authors reported that addition of an “unsure” class to account for poor-quality signals reduced the overall score, implying that quality detection is beneficial but needs to be traded off against principal classification performance. Ortiz et al. [15] exhibited inter-patient and inter-site variability when using Dynamic Time Warping (DWT). Intra-DTW features measure beat-to-beat variability between a subject, after which MFCCs yield complementary spectral information. Linear SVM works well on this feature set with 82.4 percent on the holdout test set, particularly where training and test populations vary. Linear decision boundary is easy to implement and understand but is outperformed by more expressive, non-linear kernels or tree ensembles in detecting residual nonstationary patterns. Homsi et al. [16] suggested a cascaded ensemble nested Random Forest, LogitBoost, and cost-sensitive classifier over 131 segmented time, frequency, wavelet, and statistical features. The architecture is supposed to stabilize decisions under class imbalance and recording artifacts with an achieved 84.48 percent total score, being fifth-ranked. Performance remains with reference to raw signal quality, and imbalance can constrain minority class learning, which is the appeal of introducing quality measures and calibrated thresholds. Nilanon et al. [17] addressed data sparsity by splitting long recordings into overlapping 5-second snippets and then training a CNN on inputs of MFCC and spectrogram. A voting strategy sums segment decisions to recording-level label, achieving a 0.811 score. Low sensitivity is the primary bottleneck, and the lack of low-quality exemplars forecloses “unsure” case detection, requiring data augmentation and loss functions aware of quality. Chakir et al. [18] evaluated PCG-only features against synchronized ECG and PCG features with the application of basic classifiers on a sample of 100 recordings from set A. SVM worked optimally in both settings, and the fusion timed out at 92.5 percent accuracy and 0.9505 Area Under Curve (AUC), a clear winner over PCG alone. The sample is small and from only a single subset, so the next step is to test the entire challenge distribution. Temporal evolution has been simulated by Vernekar et al. [19] using Markov chain analysis. Beats were given to 12 states with amplitude and timing, and transition probabilities were used as features. Statistical and frequency features and Markov descriptors were given to a weighted ensemble of XGBoost and ANN to achieve 81.75 percent on the validation set. Loyal noise in PCG is a challenging failure mode, since nonstationary artifacts are not entirely prevented by conventional frequency filtering. Grzegorczyk et al. [20] used a neural approach to 48 manually designed features ranked on the time axis, ordinate axis, and frequency domain. The process involved quality control and pre-rejection of clearly defective cases to prevent exposing the learner to incorrectly labeled or corrupted examples. With the use of networks and multilayer perceptrons of higher depths, the method achieved a test score of 0.79. The authors contend that metadata record location would allow for pathology-specific features and would most likely improve accuracy. Goda et al. [21] were provided with morphological features following segmentation, including S1 and S2 widths and frequency-domain summaries of systolic and diastolic durations, and wavelet envelope features following resampling. SVM classification on a fairly balanced subset yielded an average accuracy of 80.28 percent on the entire test set. The paper highlights better noise robustness and the ability to handle different recording conditions, which are still significant challenges in PCG-based screening. Table 1 shows the literature summary.

In this work, we propose a multimodal CVD classification system that fuses ECG and PCG signals by feature-based learning and adaptive probabilistic fusion. The system aims to exploit complementary diagnostic information of electrical and acoustic cardiac activity in a manner that is resilient to noise and signal variability. The system comprises three main stages: preprocessing, feature extraction, and classification. During preprocessing, ECG recordings are band-pass filtered (Butterworth, 0.5–40 Hz) with optional 50/60 Hz notch filtering and wavelet denoising (db6, level 5) for power-line and high-frequency noise removal. Baseline wander is removed using a 1.5 s moving average filter, and the signals are normalized using a robust z-score approach. R-peaks are picked up with a Pan–Tompkins-inspired algorithm for allowing rhythm and morphological feature extraction. PCG signals are resampled at 2 kHz, band-pass filtered from 25–450 Hz, and smoothed with a moving average envelope detector to highlight cardiac cycles and energy patterns. Feature extraction is customized to extract both temporal and spectral features of interest to cardiac physiology. We extract heart rate variability (HRV) parameters like mean RR interval, SDNN, RMSSD, and pNN20/pNN50, and QRS width, R-peak amplitude statistics, R/S ratios, and local energy from ECG. Spectral and wavelet-based parameters like band power ratios (0.5–5, 5–15, 15–40 Hz), energy distribution over wavelet sub-bands, spectral entropy, skewness, and kurtosis also describe the frequency-domain dynamics. PCG features include RMS energy, zero-crossing rate, spectral centroid, bandwidth, roll-off frequency, flatness, and MFCCs. Signal-quality flags, i.e., low-energy PCG or noisy ECG segments, are automatically estimated to inform downstream fusion reliability. In the classification stage, modality-specific gradient-boosted tree models (XGBoost) are trained independently for ECG and PCG features. The ECG model uses a greater positive class weight to promote sensitivity to abnormal cardiac events, and the PCG model learns complementary acoustic cues. The final decision is obtained using a confidence- and quality-aware fusion approach that adaptively weights each modality in terms of signal reliability and posterior uncertainty. Reliable ECG inputs take precedence in the fusion, with PCG contributions rising as ECG confidence declines or is high noise. The authors’ approach is developed and tested on the PhysioNet/Computing in Cardiology (CinC) Challenge 2016 database, which contains synchronized ECG and PCG recordings with normal or abnormal annotations. The varied noise conditions, sensor configurations, and patient demographics in this dataset offer a realistic test bed for measuring the robustness and generalizability of multimodal cardiac classification systems.

While there are some successful results of ECG–PCG fusion methods in terms of the classification task, there are still some weaknesses. Most of the current approaches require either the complicated network architecture and/or time-consuming fusion technique, which might complicate the use of the methods for real-time applications. Moreover, in some multimodal researches, ECG and PCG data are fused using fixed schemes without taking into account the quality or reliability of the signals in a specific recording. This aspect is important because ECG and PCG signals can be impacted by different types of noise, sensor effects, low energy, or morphology problems. In this case, the equal or fixed weighting of two modalities might degrade the decision-making process.

Therefore, the main goal of this study is not only increasing the accuracy but developing a practical, interpretable, and quality-aware ECG–PCG fusion framework. Specifically, the method under investigation uses physiologically motivated features for ECG and PCG and adjusts their weights depending on the quality and confidence levels of the signals. Such a scheme allows us to keep the diagnostic power of PCG signals and let ECG participate in the fusion process if it is reliable and informative. The key contributions of this paper include:

1. We present an adaptive ECG–PCG fusion rule that reweights modality posteriors based on signal-quality flags (e.g., ECG RR-OK, power-line noise, low-energy/flat-spectrum PCG) and posterior uncertainty, and a lightweight false-positive guard for weak ECG spectral evidence.
2. We combine wavelet-denoised ECG preprocessing with morphology, HRV, and band-power/wavelet features, augment them with PCG RMS/temporal–spectral descriptors and MFCCs, and train modality-specific XGBoost models. This maintains low computation while revealing physiologically interpretable features.
3. We train decision thresholds directly on out-of-fold posteriors to meet a user-specified specificity target, providing operating points that are retrain-free and adjustable to clinical requirements and transferable between datasets.

## 2. Materials and Methods

In this paper, a novel methodology is developed for CVD detection based on PCG and ECG signals. Both ECG and PCG features are selected for their complementing physiology that is obtained from the electrical and acoustic nature of cardiac functioning, respectively. The reason is that ECG features focus on rhythm variations, QRS morphology, and electrical spectra, whereas PCG features characterize acoustic energy, spectral content, and heart sound properties. The fusion method used in the experiment takes into account the quality of the signal, as it can vary from one ECG or PCG recording to another; hence, the weight of the two modalities changes accordingly.

**Figure 1:**
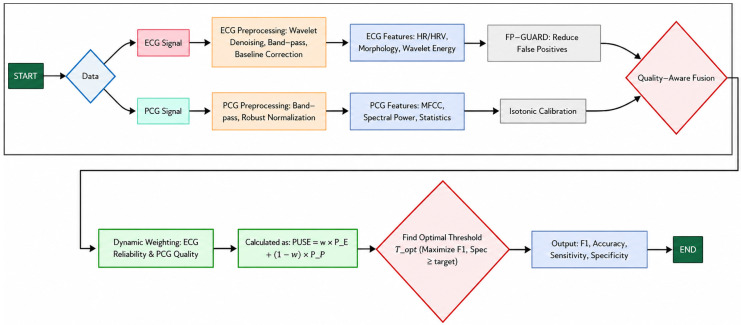
The flowchart of the proposed methodology.

Figure 1 shows the flowchart of the proposed methodology. From the Observation of Figure 1, the approach uses a multimodal fusion methodology that combines ECG and PCG recordings to promote the credibility of cardiovascular disease diagnosis. The process starts by collecting data from the PhysioNet 2016 Challenge dataset and then preprocesses each modality independently. The ECG signals are wavelet-based denoised, band-pass filtered, and baseline corrected to eliminate motion artifacts and low-frequency drift, and the PCG signals are band-pass filtered and robustly normalized to eliminate the amplitude variability. Features that are applicable across both modalities are extracted after the preprocessing. Features of ECG include HR, HRV, morphology, and wavelet energy, while those of PCG are obtained via MFCCs, spectral power, and statistics.

These features are mapped to probabilistic estimates by individual classifiers per modality. On the ECG side, a guard against false alarms suppresses suspect scores to prevent alarms; on the PCG side, isotonic calibration tightens the precision of the prediction probabilities. These two calibrated results are blended into one by a quality-guided fusion step that dynamically tips the influence balance to the modality that is assumed to be most reliable for that record, based on ECG reliability measures and PCG quality measures. Rather than a formula, the combined score can be thought of as confidence-weighted sum of the probabilities of the ECG and the PCG, with weight increasing as the modality is cleaner and more consistent. A final operating threshold is selected to maximize F1 subject to a target specificity, and the system reports F1, accuracy, sensitivity, and specificity, showing complementary ECG and PCG evidence leads to more accurate and robust abnormality detection.

### 2.1. Preprocessing

#### 2.1.1. Robust Normalization and Envelope

The input PCG and ECG signals are initially normalized according to a robust z-scoring procedure based on the sample median and the Gaussian-consistent median absolute deviation (MAD*). For a discrete signal x[n], the normalized sequence is:

(1) $$z\left[n\right]=\frac{x\left[n\right]-median(x)}{{MAD}^{*}},\;\;\;{MAD}^{*}=\frac{median(\left|x\left[n\right]-median\left(x\right)\right|)}{0.6745}$$

A short-term amplitude envelope is then formed by a symmetric moving average of ∣[*n*]∣ over a window of K samples,

(2) en=1K∑i=n−K2n+K2z[i]

which serves as a robust proxy for local energy and facilitates downstream feature computation [22,23].

#### 2.1.2. PCG Preprocessing

Heart-sound content is isolated by band-limiting the PCG to 25–450 Hz via cascaded high-pass and low-pass filtering; optional pre-emphasis enhances high-frequency transients relevant to valve closures:

(3) $$x_{bp}\left[n\right]=B_{lp}\left(450\right)\circ B_{hp}\left(25\right)\left\{x_{pcg}\left[n\right]\right\},\;\;\;x_{pre}\left[n\right]=x_{bp}\left[n\right]-\alpha x_{bp}\left[n-1\right],\;0<\alpha <1$$

The normalized PCG and its envelope *e*pcg[*n*], which can be computed in (2) with a ∼60 ms window, are used for feature extraction [24,25,26,27,28,29,30].

#### 2.1.3. ECG Preprocessing

Electrocardiographic activity is confined to 0.5–40 Hz using band-pass filtering; mains interference at 50/60 Hz is attenuated by notch filtering. Remaining high-frequency artifacts are reduced by wavelet shrinkage with a Daubechies-6 (db6) multiresolution decomposition and the universal soft threshold,

(4) $$T=\sigma \sqrt{2lnN},\;\;\;\;\;\;\;\;\;\;\;\;\sigma =\frac{median(\left|D_{1}\right|)}{0.6745}$$

where *D*1 denotes the finest-scale detail coefficients and *N* the signal length [31,32]. Low-frequency baseline wander is removed by subtracting a long moving average of duration *τ* ≈ 1.5 s:

(5) xblrn=xfn−1Kτ∑i=n−Kτ2n+Kτ2xf[i]

after which a mild saturation confines outliers while preserving morphology,

(6) $$x_{norm}\left[n\right]=tanh\left(\frac{x_{blr}\left[n\right]-\mu }{\sigma }\right)$$

#### 2.1.4. PCG Feature Set

From PCG waves, we extracted various time domain key features. Time-domain descriptors include the root-mean-square energy and the zero-crossing rate,

(7) $$RMS=\frac{1}{N}\sum_{n=1}^{N}x{\left[n\right]}^{2},\;ZCR=\frac{1}{N-1}\sum_{n=1}^{N-1}1\left\{x\left[n\right]x\left[n+1\right]<0\right\}$$

Welch’s power spectral density $P_{x}\left(f\right)$ yields bandpowers and spectral shape statistics,

(8) $$P_{20-150}=\int_{20}^{150}P_{x}\left(f\right)df,\;\;\;\;P_{150-400}=\int_{150}^{400}P_{x}\left(f\right)df,\;\;\;\;$$

The spectral centroid (C) represents the weighted mean frequency of the power spectral density *P*(*f*), quantifying the center of mass of the spectrum; the spectral bandwidth (BW) measures the root mean square deviation of the spectrum around the centroid, describing its frequency dispersion; and the spectral flatness (SF) is the ratio of the geometric to the arithmetic mean of *P*_*x*_(*f*), indicating how noise-like (flat) or tonal (peaked) the spectrum is:

(9) $$C=\frac{\sum_{f}fP_{x}\left(f\right)}{\sum_{f}P_{x}\left(f\right)},\;\;\;\;BW=\frac{\sum_{f}{\left(f-C\right)}^{2}P_{x}\left(f\right)}{\sum_{f}P_{x}\left(f\right)},\;\;\;SF=\frac{e^{\frac{1}{N_{f}}\sum_{f}lnP_{x}\left(f\right)}}{\frac{1}{N_{f}}\sum_{f}P_{x}\left(f\right)}$$

capturing centroid, bandwidth, and spectral flatness, respectively [24,25,26]. Cepstral structure is summarized by the first two moments of MFCC $c_{k}\left[t\right]$:

(10) $$c_{k}\left[t\right]=\;\sum_{m=1}^{M}\ln S\left[m,t\right]\cdot \cos\left[\;\left(\frac{\pi \;k}{M}\right)\cdot \;\left(m\;-\;0.5\right)\right]\;\mu_{k}\;=\;{mean}_{t}\left(\;c_{k}\left[t\right]\right),\;\;\;\sigma_{k}\;=\;{std}_{t}\left(\;c_{k}\left[t\right]\right)$$

where $S\left[m,t\right]$ is the Mel-scaled filter bank energy at frame t. And $\mu_{k}\;$ and σk are used to summarize their time evolutions, respectively.

#### 2.1.5. R-Peak Detection and HRV Statistics

A Pan–Tompkins-style sequence (band-pass, derivative, nonlinearity, moving integration) identifies R-peaks $p_{j}$ [33]. After excluding physiologically implausible intervals, the RR series and canonical variability indices are computed:

(11) $${RR}_{j}\;=\frac{\left(p_{j+1}-\;p_{j}\right)}{{f_{s}}^{ECG}},\;\;\;\;HR\;=\;\frac{60}{mean\left(RR\right)}$$

(12) $$SDNN=std\left(RR_{ms}\right),RMSSD=\sqrt{mean\left({\left({RR}_{j+1,ms}-{RR}_{j,ms}\right)}^{2}\right)}$$

(13) $$pNN20=mean\left(\left|\Delta RR_{ms}\right|>20\right),pNN50=mean\left(\left|\Delta RR_{ms}\right|>50\right)$$

QRS width *w*QRS is estimated by peak width at half height and summarized by mean and standard deviation.

#### 2.1.6. ECG Morphology, Spectral Bands, and Wavelets

Within short windows centered at R, morphology is characterized by the average slope, the R/S amplitude ratio, and local energy,

(14) $$Slope=\frac{1}{M}\sum_{i}\frac{\left(x\left[i+1\right]-x\left[i\right]\right)}{\Delta t},RS-ratio=\frac{\left|R\right|}{\left(\left|S\right|+\varepsilon \right)},E_{local}=\sum_{i}x{\left[i\right]}^{2}$$

Spectral content is summarized by Welch bandpowers in 0.5–5, 5–15, and 15–40 Hz and their normalized ratios,

(15) $$S=P_{0.5-5}+P_{5-15}+P_{15-40},\;\;\;\;\;\;\;\;r_{1}=\frac{P_{0.5-5}}{S},r_{2}=\frac{P_{5-15}}{S},r_{3}=\frac{P_{15-40}}{S}$$

A db6 level 5 wavelet decomposition yields approximation *A*5 and details (*k* = 1, …, 5); their energies and ratios are;

(16) $$E_{A}=\sum A_{5}^{2},E_{Dk}=\sum {Dk}^{2},S_{w}=E_{A}+\sum_{k=1}^{5}E_{Dk},r_{A}=\frac{E_{A}}{S_{w},},\;r_{Dk}=\frac{E_{Dk}}{S_{w}}$$

Let $c_{i}$ denote the concatenated wavelet coefficients and let $p_{i}=|c_{i}{|}^{2}/\sum_{j}{|cj|}^{2}$ denote the normalized wavelet energy ratio. Wavelet entropy and shape-related descriptors are then computed as follows:

(17) $$H\;=\;-\sum_{i}\;p_{i}\ln\left(p_{i}\right),\;\;\;\;skew\left(c\right),\;\;\;\;kurt\left(c\right)$$

which captures multiscale complexity and distributional asymmetry [34]. In equation 17, the skew(c) and kurt(c) show the skewness and kurtosis functions.

#### 2.1.7. ECG False-Positive Guard

To mitigate spurious positives from weak or morphologically atypical ECG segments, an out-of-fold (OOF) reliability rule is defined [35]. Let *q*0.20 denote the 20th percentile of the OOF distribution of *P*15–40. The guard event is;

(18) $$G=\left\{\;P_{15-40}<q_{0.20}\right\}\cap \;\left\{\;r_{D4}<0.08\;\right\}\cap \;\left\{\;\bar{RS-ratio\;}<1.2\;\right\}$$

under which the ECG posterior is attenuated,

(19) $$p_{ECG.guard}=\left(\begin{array}{c} {0.65p}_{ECG},\;\;\;\;\;\;\;\;\;\;\;G\;true\\ p_{ECG},\;\;\;\;\;\;\;\;\;\;\;\;\;\;\;\;\;\;\;\;otherwise \end{array}\right.$$

#### 2.1.8. Modality-Wise Classifiers

For each modality, a probabilistic classifier with logistic link is trained on its feature vector *ϕ* [36,37]. The posterior takes the form

(20) $$p=\sigma \left(f\left(\varphi \right)\right)=\frac{1}{\left(1+\exp\left(-f\left(\varphi \right)\right)\right)}$$

where *f*(⋅) denotes an additive ensemble of decision functions. Parameters are learned by minimizing a weighted logistic loss with regularization,

(21) $$L=\sum_{i}w_{i}\left[-y_{i}\ln\left(p_{i}\right)-\left(1-y_{i}\right)\ln\left(1-pi\right)\right]+\Omega \left(f\right)\;w_{pos}=\max\left(4.0,\;\frac{N_{neg}}{\max\left(1,\;N_{pos}\right)}\right)$$

which addresses class imbalance and controls model complexity [38].

#### 2.1.9. Quality-Aware Dynamic Fusion

Final decisions are based on a convex combination of the guarded ECG posterior and the PCG posterior [39],

(22) $$p_{fuse}=w_{eff}\cdot p_{ecg,guard}+\left(1-w_{eff}\right)\cdot p_{pcg}$$

The effective ECG weight incorporates a base term, a PCG quality bonus, and a confidence modulation,

(23) $$c={\left(2\cdot \left|p_{ecg,guard}-0.5\right|\right)}^{2}\;ẇ=\min\left(w_{base}+0.15\cdot 1_{pcg,bad},\;1\right)\;w_{eff}=\min\left(ẇ\cdot c,\;0.75\right)$$

and is set to zero when rhythm detection is unreliable or the ECG is noisy, in line with reliability-weighted sensor fusion principles [40,41].

#### 2.1.10. Threshold Selection Under a Specificity Constraint

Let $\hat{y}\left(T\right)=\;1\{p\geq T\}$ denote the decision at threshold *T*. The operating point is determined as follows [42]:

(24) $$T^{*}\;=\arg\underset{T\in \left[0,1\right]}{\max}F_{1}\left(T\right)\;\;subject\;to\;\;\;Spec\left(T\right)\geq \;S_{min}$$

With confusion-matrix counts *TP*, *TN*, *FP*, *FN*, the performance indices are:

(25) $$Sens\;=\;\frac{TP}{\left(TP\;+\;FN\right)}\;Spec\;=\;\frac{TN}{\left(TN\;+\;FP\right)}\;Prec\;=\;\frac{TP}{\left(TP\;+\;FP\right)}\;Acc\;\;=\frac{\left(TP\;+\;TN\right)}{\left(TP\;+\;TN\;+\;FP\;+\;FN\right)}\;F1=\;2\cdot \frac{Prec\cdot Sens}{\left(Prec\;+\;Sens\right)}$$

If the constraint is infeasible, a penalized Youden objective provides a fallback [43,44],

(26) $$J_{\lambda \left(T\right)}=\left(Sens\left(T\right)+Spec\left(T\right)-1\right)-\lambda_{spec}\cdot {\left[\tau_{spec}-Spec\left(T\right)\right]}_{+}-\lambda_{sens}\cdot {\left[\tau_{spec}-Sens\left(T\right)\right]}_{+}$$

whose maximizer T† balances sensitivity and specificity under minimum-performance penalties [33,35,36,43].

## 3. Experimental Works and Results

Calculations were made in Python 3.11 with torchaudio for audio I/O and MFCC extraction, SciPy for signal processing (zero-phase Butterworth band-/notch filtering, Welch spectra, Savitzky–Golay smoothing, peak widths), scikit-learn for scaling, cross-validation, metrics, and isotonic calibration, and XGBoost for gradient-boosted logistic classification. The database is the PhysioNet/CinC 2016 heart sound corpus; for every recording, the PCG waveform (WAV) is always read, and, when present, the ECG is read from WFDB. PCG preprocessing includes windowing, band-pass filtering to 25–450 Hz, optional pre-emphasis, robust median–MAD normalization, and a ~60 ms envelope. ECG preprocessing comprises windowing, 0.5–40 Hz band-pass filtering, 50/60 Hz notch suppression, db6 level 5 wavelet shrinkage with universal threshold, 1.5 s baseline removal, robust normalization, and gentle saturation. PCG features consist of RMS, zero-crossing rate, Welch band powers (20–150 and 150–400 Hz), spectral centroid/bandwidth/flatness, and MFCC means and standard deviations. ECG features include R-peak detection via a Pan–Tompkins-type pipeline, HRV measures (HR, SDNN, RMSSD, pNN20/pNN50), QRS width measures, local morphology (R amplitude, mean slope, R/S ratio, local energy), Welch bandpowers (0.5–5/5–15/15–40 Hz) and ratios thereof, wavelet energies/ratios and entropy, skewness, and kurtosis. Quality measures per record are also calculated. Stratified K-fold cross-validation is used for evaluation. Features are normalized with a robust scaler by default (standardization is also an option), and two XGBoost classifiers are trained separately on PCG and ECG. The ECG model has more aggressive class weighting and a lower learning rate to prefer sensitivity. Fold-wise isotonic regression is the optional posterior calibration step. A quality-conscious fusion convexly combines the PCG and ECG posteriors with a dynamic ECG weight that is only increased when RR detection is valid, the ECG is noiseless, and ECG confidence is high; the weight is clipped to prevent domination and is boosted when PCG quality is low. Furthermore, a false-positive guard down-weights ECG scores under low-power/morphology conditions obtained from out-of-fold statistics. The decision threshold was chosen on a per-split basis through optimization of F1 score under a minimum specificity constraint, and we report accuracy, sensitivity, specificity, and F1 score per fold, together with the final out-of-fold summary computed from the concatenated held-out predictions.

Additional implementation details were added to improve reproducibility. In the final implementation, 53 PCG features and 44 ECG features were extracted for each recording. The PCG XGBoost classifier was trained with *n*_estimators = 2000, max_depth = 4, learning_rate = 0.02, subsample = 0.90, colsample_bytree = 0.90, reg_lambda = 1.0, reg_alpha = 0.0, min_child_weight = 1, and gamma = 0.0. The ECG XGBoost classifier used *n*_estimators = 3400, max_depth = 3, learning_rate = 0.010, subsample = 0.85, colsample_bytree = 0.85, reg_lambda = 1.5, reg_alpha = 0.3, min_child_weight = 14, gamma = 2.5, and class weighting through scale_pos_weight. Early stopping was applied with 100 rounds. When enabled, posterior probabilities were calibrated using isotonic regression. The decision threshold was searched between 0.05 and 0.95, and the fusion weight was searched between 0 and 1 using 41 candidate values.

### 3.1. Dataset

**Figure 2:**
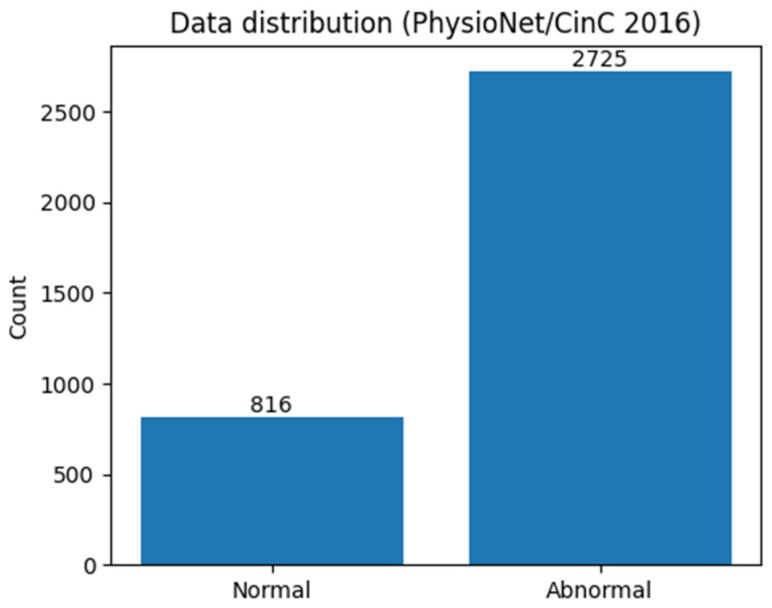
The distribution of data Normal and Abnormal cases.

The PhysioNet/Computing in Cardiology (CinC) Challenge 2016 database [29] has been used in this study. The database was obtained from the 2016 PhysioNet/CINC challenge, which focused on the classification of heart sounds and was available on the PhysioNet website. The PhysioNet/Computing in Cardiology Challenge 2016 includes ECG and PCG signals registered from a heterogeneous sample of the population with equal gender distribution and an age range between 18 and 85 years. It is expected that this age range will contribute to the generalization of the model for different cardiac profiles. The datasets, named from training-a to training-f and validation, were gathered by PhysioNet from a number of research institutions. Specifically, the training-a subset, consisting of 409 samples, was contributed by MIT and comprises a combination of ECG and PCG signals. Among these samples, 405 contain both ECG and PCG signals, while the remaining 4 have just PCG signals. There are 113 normal signals and 292 abnormal signals in this dataset. Figure 2 shows the histogram of the normal and abnormal classes. All available recordings from the training-a to training-f and validation subsets were considered. Records were included when the corresponding PCG waveform file was available. When the matched ECG WFDB files were present, ECG information was also used; otherwise, the recording contributed only PCG-based information. No additional patient-level exclusion criterion was applied beyond the availability of the required signal files. As seen in Figure 2, there are 816 and 2725 normal and abnormal samples in the dataset.

**Figure 3:**
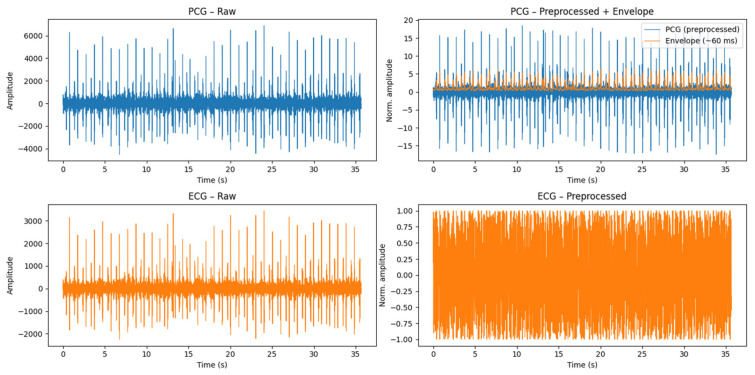
A sample of raw and preprocessed PCG (a0001.wav) and ECG (a0001.dat) signals.

All the signals were initially recorded with a sampling rate of 44.1 kHz, but afterwards, they were resampled at 2000 Hz as a post-processing step. Figure 3 shows a raw PCG and ECG signal and their preprocessed forms.

**Figure 4:**
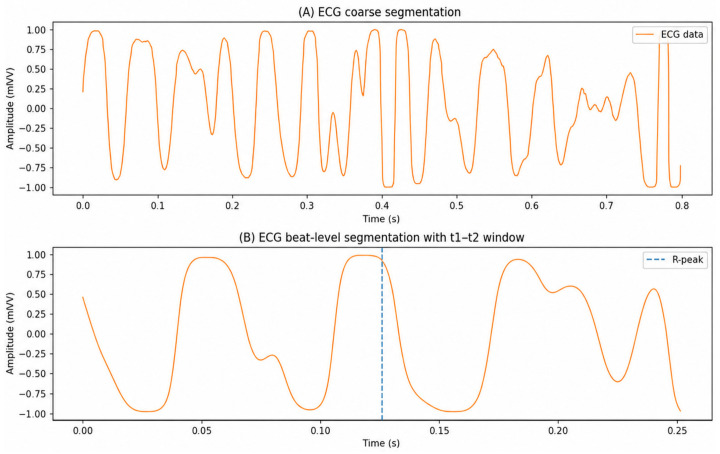
ECG coarse segment and ECG beat level segmentation with t1-t2 window.

Figure 4 shows both planes of the ECG segmentation employed for delimiting cardiac activity patterns. For Panel A, the coarse segmentation gives a consistent segment of the normalized ECG trace over a small temporal window (≈0.8 s), bounding the global rhythm and shape of several beats. This operation provides a crude approximation of the signal’s amplitude variation and periodicity. In Panel B, the focus is on beat-level segmentation, in which one beat is isolated.

The R-peak recognized (a dotted vertical line) indicates the primary depolarization episode of the heart’s cycle. Such higher-level segmentation is useful for precise alignment of future beats and facilitating an analysis of intervals such as t_1_–t_2_, time windows around each beat.

### 3.2. Results

In this study, the out-of-fold validation was performed based on the approach of stratified 10-fold cross-validation. In each run, nine folds are used as the training data, whereas one fold acts as the unseen hold-out data. All data-driven processes such as scaling, classifier training, probability calibration, fusion weightings, and threshold setting are carried out only based on the training folds. The hold-out fold is used only to predict. Predictions based on posterior probabilities generated from each hold-out fold were noted. When the procedure for all 10 folds was completed, all the predictions were put together to obtain the final out-of-fold validation results.

Table 2 shows the entire 10-fold profile with clear-cut trends and concrete statistics. The ECG model keeps its sensitivity very high in every fold from 96.3% to 98.9%, but its specificity is always poor and unreliable from 24.7% to 45.1%. This reduces accuracy to the interval 79.9% to 85.3% and yields F1 between 88.1% to 91.1%. These statistics evince a strong inclination to label abnormal cases but permit many normal cases to be marked incorrectly. PCG model has good behavior with well-balanced behavior. F1 ranges from 95.9% to 98.4%, and accuracy ranges from 93.5% to 97.5%. Specificity is much greater than ECG alone, with values mostly between the mid-80s to the low 90s, and one lower outlier of 75.3% in fold eight. Sensitivity is very strong, ranging from 96.0% to 99.3%. This stability suggests that acoustic features in heart sounds hold discriminative clues for both classes.

Blending of ECG and PCG combines the strengths of both, and it does it across all folds. F1 stays comfortably within 96.0% to 98.7%, and accuracy stays between 93.8% and 98.0%. Specificity is much better than ECG alone and is consistently high, from 81.5% to 95.1%, while sensitivity stays close to ceiling from 96.3% to 98.9%. Highest gains are observed where ECG specificity is low. For fold two, specificity improves from 30.5% with ECG to 84.1% with fusion, and F1 also improves from 88.7% to 96.6%. For fold three, specificity improves from 32.9% to 87.8%, and F1 improves from 89.7% to 97.3%. For fold nine, specificity improves from 24.7% to 86.4%, and F1 improves from 88.1% to 96.7%. Even when PCG performs well already, fusion continues with incremental but consistent improvement. In fold one, fusion achieves the best figures, with 98.4% F1, 97.5% accuracy, 92.7% specificity, and 98.9% sensitivity. In fold six, fusion achieves the overall best figures: 98.7% F1, 98.0% accuracy, 95.1% specificity, and 98.9% sensitivity.

Table 3 combines average performance with fold variation and validates such trends with ultimate out-of-fold results. The ECG model is highly sensitive at 97.2% ± 0.8% with a corresponding OOF of 97.8%, but its low on specificity at 34.1% ± 5.4% with an OOF of 32.6%, which brings accuracy to 82.7% ± 1.6% with an OOF of 82.7% while keeping the F1 score at 89.6% ± 0.9% with an OOF of 89.7%. The PCG model is much better balanced. It reaches an average F1 score of 96.8% ± 0.7% with an OOF of 96.8% and accuracy of 95.0% ± 1.2% with an OOF of 95.0%. Specificity increases to 86.0% ± 5.3% with an OOF of 86.0%, while the sensitivity remains high at 97.7% ± 1.1% with an OOF of 97.7%. The quality-aware fusion further optimizes the operating point without compromising recall. The average F1 score is increased to 97.2% ± 0.9% with an OOF of 97.1% and accuracy of 95.6% ± 1.4% with an OOF of 95.6%. Specificity is increased to 88.6% ± 4.7% with an OOF of 88.5%, while sensitivity remains high at 97.7% ± 0.8% with an OOF of 97.7%. The small differences between the fold averages and the OOF values indicate stable generalization. ECG, in real life, identifies nearly all abnormal cases but mistakenly classifies a considerable number of normal cases. PCG remedies much of this imbalance by increasing specificity without sacrificing sensitivity.

### 3.3. Ablation Study

In this work, an extensive ablation analysis is carried out to quantify the contribution of each signal modality and each preprocessing component in the proposed ECG-PCG fusion framework. Specifically, we conducted a systematic evaluation of various configurations derived from the full fusion model by selectively disabling individual components such as wavelet-based ECG denoising, quality-aware fusion guard, and MFCC-based PCG features while also comparing different scaling schemes (Robust vs. Standard) and calibration modes on the PhysioNet/CinC 2016 heart sound classification dataset using a 10-fold cross-validation protocol. All ablations were performed using identical data splits, random seeds, and optimization settings. Results across folds are summarized with mean and standard deviation for the F1 score, accuracy, specificity, and sensitivity alongside OOF metrics and optimal thresholds. This structured ablation enabled the clear attribution of the performance improvements to the respective design choices; in particular: the role of ECG wavelet processing, the value of PCG spectral features, and the adaptive fusion weighting mechanism. All these prove the robustness and interpretability of the proposed 10-fold ECG-PCG fusion pipeline.

Table 4 shows the quantitative comparison in the ablation studies by removing/modifying certain components of the ECG–PCG fusion framework. Among the single modality models, it is obvious that the PCG-only configurations outperform the ECG-only ones by a large margin: a 96.8% and 95.4% F1 score for the variant with and without MFCC, respectively, while ECG-only stays around 80.5%. Similarly, large gaps are observed in accuracy: about 95.0% versus 76.6%. For sensitivity, PCG-only with MFCC achieves as high as 97.7%, way higher than the 86.7% obtained by the ECG-only, and for specificity, it reaches 86.0% against the mere 42.8% of ECG-only. When the two modalities are fused, improvements persist across all metrics. The quality-aware fusion model records 96.8% F1 and 95.1% accuracy. The simple average-based fusion slightly improves to a 97.2% F1 score and 95.7% accuracy, with its highest specificity of 88.2%. The best performing overall should be the calibrated fusion variant: a 97.3% F1 score, 95.8% accuracy, 98.2% sensitivity, and 87.9% specificity, indicating that calibration helps in fine-tuning the decision boundary for optimal trade-offs between sensitivity and specificity.

A PCG-only configuration is expected to perform well because the PhysioNet/CinC 2016 dataset focuses primarily on heart sound classification. Therefore, PCG features provide direct and strong discriminatory information in distinguishing between normal and abnormal cases. However, the inclusion of ECG in the model is not solely intended to significantly increase accuracy under ideal PCG conditions. ECG provides complementary information regarding rhythm variability, QRS morphology, and the electrical activity of the heart. This information can particularly support the decision-making process when the PCG recording is noisy, low-energy, or unclear.

Furthermore, the proposed fusion strategy does not assign fixed or equal weights to ECG and PCG. The contribution of each modality to the final decision is dynamically adjusted according to signal quality and predictive confidence level. Thus, ECG is used as a supporting resource only when it is reliable and informative; when PCG quality is high, the model relies heavily on PCG information. This explains why the fusion model provides a numerically limited improvement compared to PCG-only classification. However, it also demonstrates that it offers a more flexible and quality-sensitive multimodal decision framework.

Despite extracting an extensive range of ECG and PCG features as a means of capturing temporal, spectral, morphological, and rhythmic data, no explicit manual feature selection or dimensionality reduction was performed before performing classification tasks. However, feature selection is performed implicitly due to the nature of XGBoost as a regularized and tree-based algorithm, where any feature which does not significantly contribute towards making decisions is less likely to affect the final decision. Explicit feature selection can help in simplifying the model and making it more interpretable. Therefore, evaluation of small feature sets through feature importance rankings and wrappers is seen as a valuable area of future research.

It is also noted that, in some folds, the PCG-only classifier achieved a slightly higher F1 score than the combined ECG-PCG classifier. The reason behind such results lies in the fact that the main purpose of the PhysioNet/CinC 2016 dataset is heart sound classification, while PCG features contain high discriminating power. The fusion method described in this paper is not meant to outperform the PCG-only classifier in each individual fold. It is meant to provide an adaptive and quality-aware multimodal framework where ECG can add its value whenever possible.

In addition, some ECG-only variants produced identical performance values in the ablation study. This indicates that removing the ECG guard, wavelet denoising, or changing the scaler did not measurably alter the final ECG-only predictions under the present dataset and thresholding strategy. Therefore, these components should not be interpreted as independently improving ECG-only classification performance; rather, they mainly serve as signal conditioning and robustness-control steps.

## 4. Discussions

This paper presents a compact, feature-driven fusion framework for heart sound classification that effectively leverages the complementary information in PCG and ECG while maintaining a lightweight and reproducible pipeline. The design centers around physiologically motivated descriptors: PCG spectral statistics and MFCC summaries capture energy patterns representative of murmurs, whereas ECG morphology, band-power ratios, and rhythm variability provide cues regarding conduction and rate irregularities. Two XGBoost classifiers work in tandem upon modality-specific features, and a simple quality-aware fusion combines their probabilities, providing safeguards for noisy ECG segments and low-energy PCG cases. The training and evaluation follow under a stratified 10-fold protocol with identical seeds and splits in order to ensure comparability across variants fairly; thresholds are selected under a target specificity to reflect realistic screening needs.

Table 5 presents a comparative evaluation of previous studies using the same PhysioNet/CinC 2016 dataset with the proposed quality-sensitive fusion method. As shown in Table 5, some previous studies achieved high accuracy values on this dataset. For example, among single-modality studies, Chowdhury et al. (2018) [12] achieved 97.1% accuracy with a five-layer DNN structure using only PCG signals. In contrast, Cheng et al. (2021) [39], based only on ECG signals, reported 89.3% accuracy. This indicates that ECG signals alone may provide limited information in identifying heart sound anomalies. Multimodal approaches using both ECG and PCG signals have generally improved classification performance and decision robustness. For example, Li et al. (2022) [40] achieved 96.1% accuracy with a BiLSTM-CNN architecture, while Chakir et al. (2020) [18] and Singh et al. (2021) [41] achieved accuracy values of 92.5% and 93.1%, respectively. Similarly, Chandrasekhar et al. (2025) [9] reported 97.3% accuracy with the PJM-DJRNN model.

Compared to these studies, the proposed quality-sensitive ECG–PCG fusion method achieved an overall accuracy of 95.6%. While this value does not exceed the highest accuracy value reported in the literature, the main contribution of the proposed method is not only to provide a large increase in the accuracy metric. The added value of the study is that it offers a lightweight, interpretable ECG–PCG fusion framework that directly incorporates signal quality into the decision-making process. Unlike more complex deep-learning architectures, the proposed method uses physiologically interpretable ECG and PCG features and dynamically combines modality-specific predictions for each recording based on signal reliability and posterior confidence level. Thus, the impact of noisy or less reliable signal components on the final decision is reduced, while the contribution of the more informative modality is increased.

Therefore, the proposed method should be evaluated not only in terms of overall accuracy but also in terms of practical usability, computational efficiency, interpretability, and the balance between sensitivity and specificity. The results show that the quality-sensitive fusion strategy increases specificity while maintaining a high level of sensitivity compared to ECG-only classification. This is particularly important in screening applications, as in such systems, it is clinically valuable not only to detect abnormal cases but also to reduce false positive decisions and unnecessary referrals. Therefore, the proposed method can be considered a practical alternative that offers competitive performance with a simpler, faster, interpretable, and quality-aware structure, rather than a complex model aiming for the highest accuracy value.

From a clinical perspective, the proposed framework may be useful as a screening-support tool rather than as a stand-alone diagnostic system. Its high sensitivity indicates that it can detect most abnormal cases, while the improved specificity may help reduce false-positive decisions and unnecessary referrals. The lightweight feature-based structure and short running time also make the method potentially suitable for real-time or resource-limited cardiac screening settings. However, clinical use would require further validation on independent datasets and evaluation by medical experts.

### 4.1. Running Time

We further investigated our model in terms of running time to assess its computational efficiency and practicality. The runtime evaluation was performed on a workstation equipped with an Intel Core i7 CPU, 64 GB system RAM, and an NVIDIA GeForce RTX 3090 GPU with 24 GB GPU memory and CUDA support. The reported runtime values were obtained on this hardware configuration. The run time results indicate that the proposed method operates with high computational efficiency and has highly consistent behavior. It took only 66 s to run a complete 10-fold cross-validation, thus reaching an average of approximately 2.2 s per fold. Single-fold run times were very stable at around 2 s, with minor variations up to 3 s for a few folds. This small variability is due to background system scheduling and input/output buffering rather than variations in model performance. The narrow range of times suggests predictable computational demand and efficient use of resources across folds. The stability of computational demand makes it well-suited for iterative experimentation, ample parameter tuning, and for deployment scenarios where retraining or adaptation at fast rates is necessary. With compact features and a lightweight classifier based on XGBoost, this approach enjoys a good balance of accuracy and run time, showing that it maintains speed and reproducibility even on repeated validation cycles.

### 4.2. Explainability Analysis

**Figure 5:**
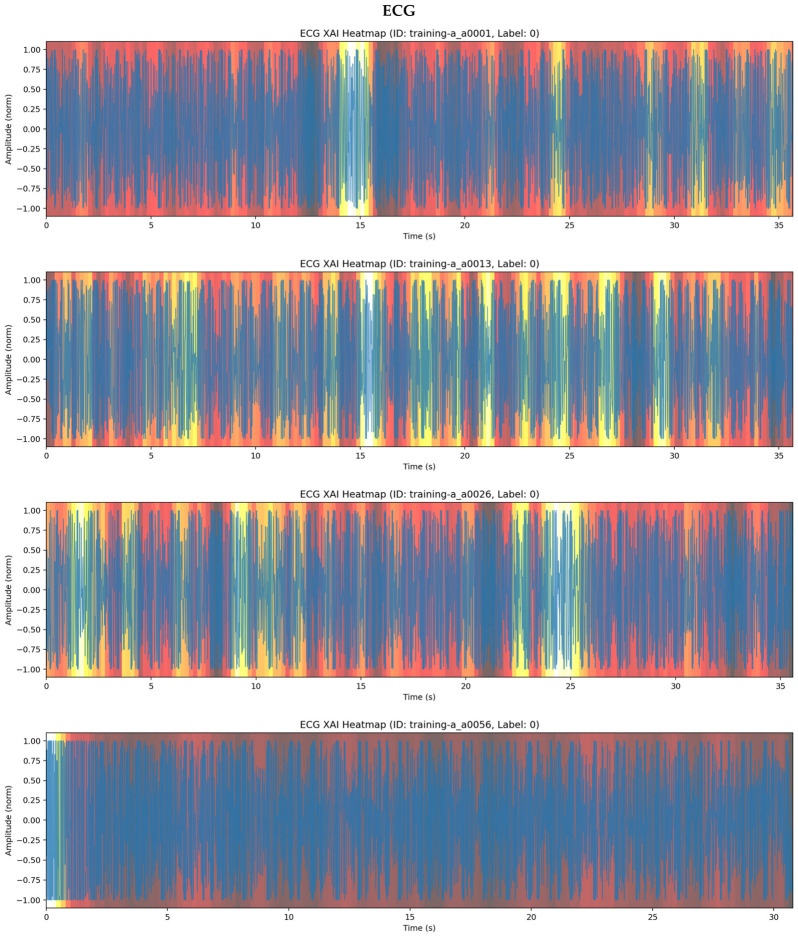

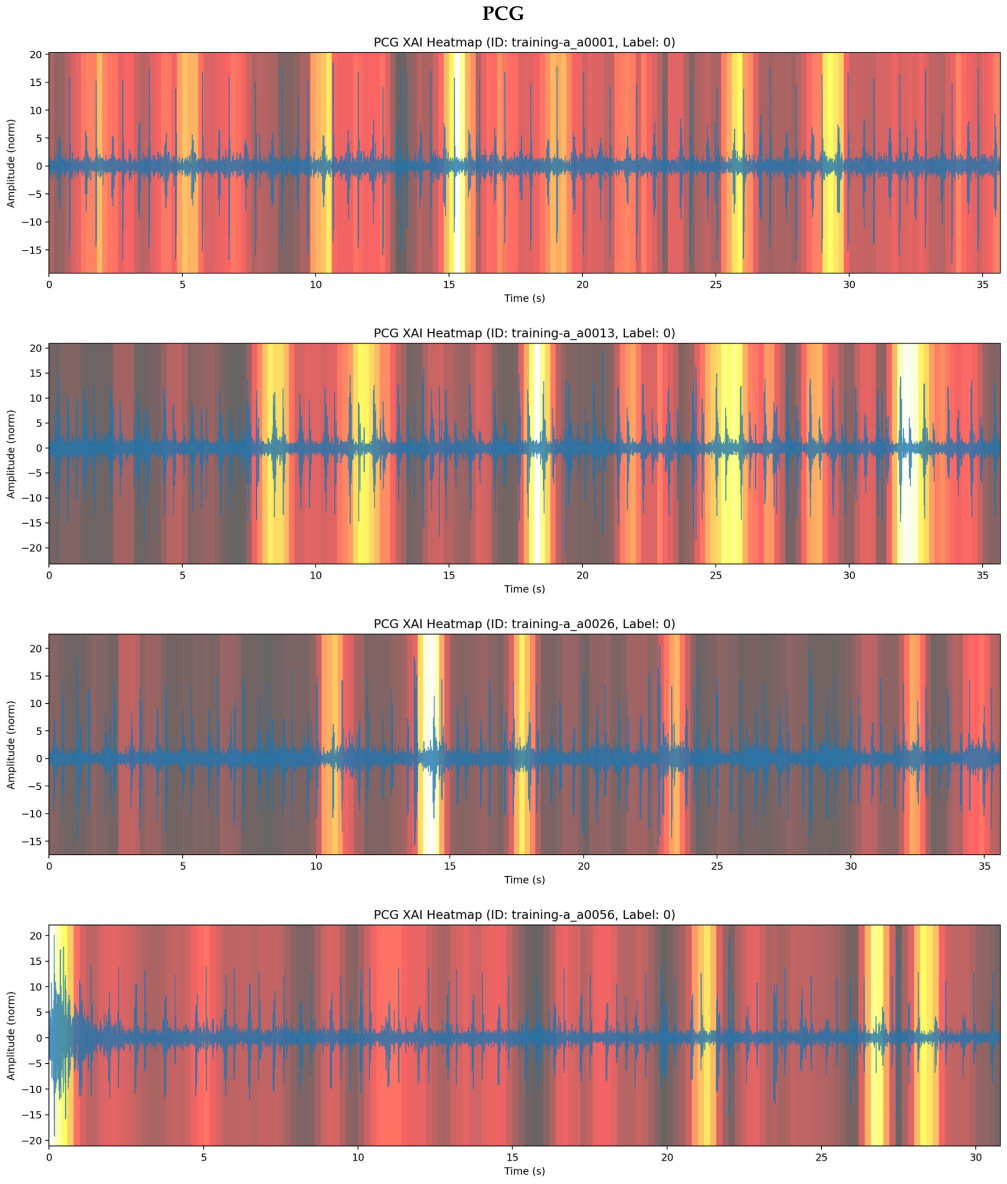
Explainability analysis on normal class ECG and PCG signals.

**Figure 6:**
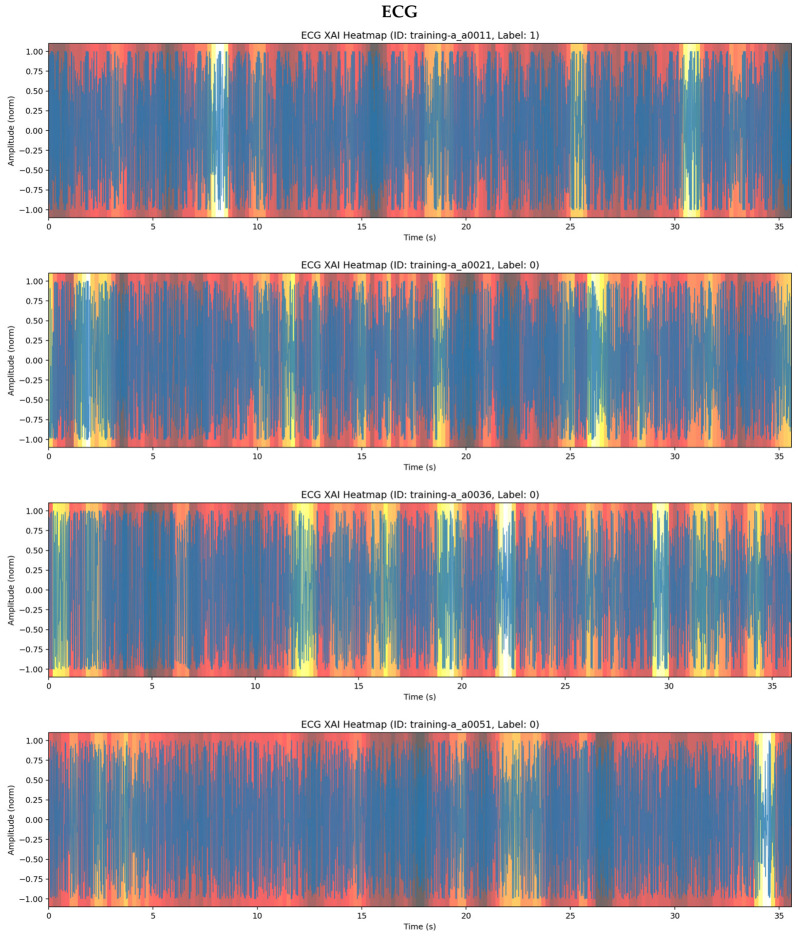

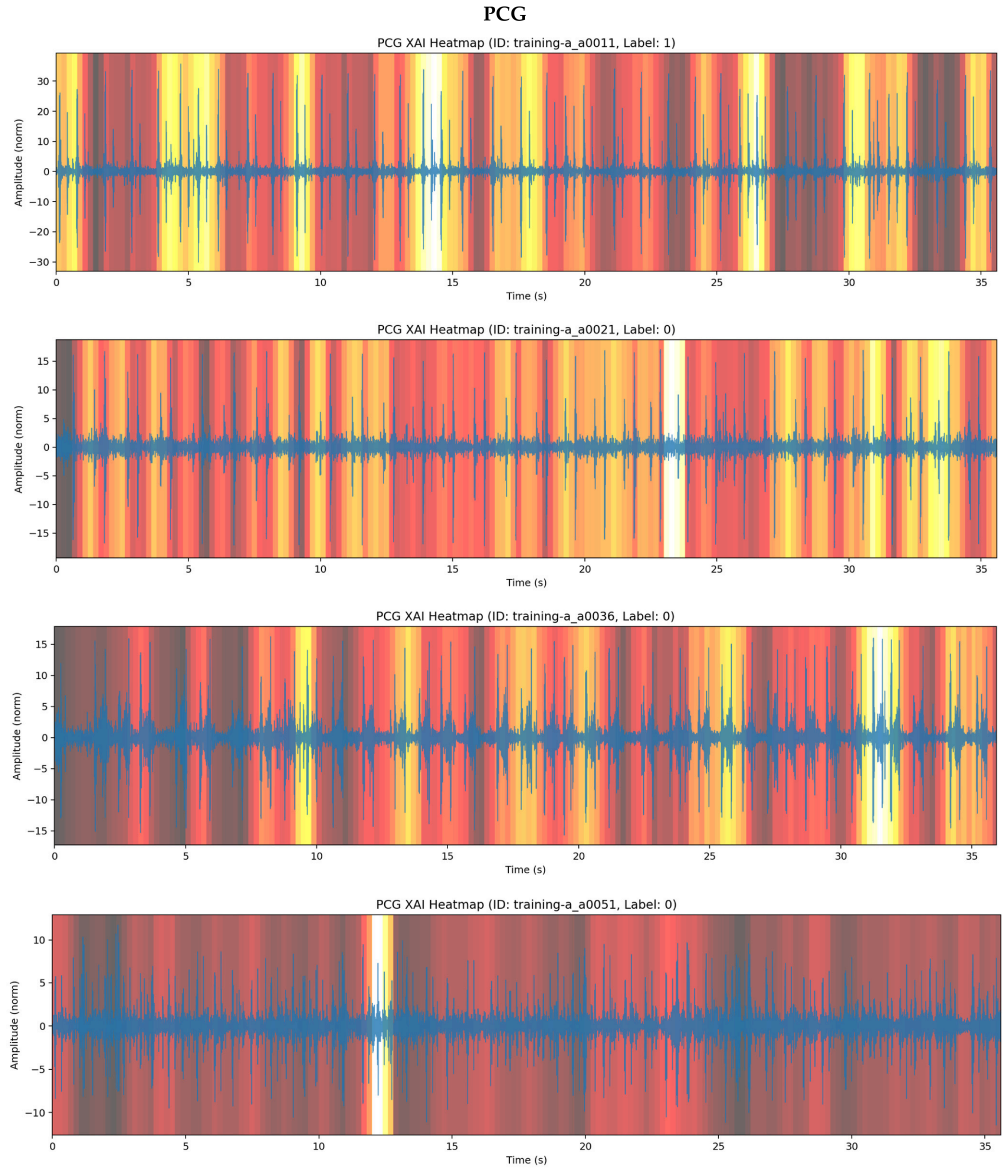
Explainability analysis on abnormal class ECG and PCG signals.

The explainability process was designed to provide a time-aligned, transparent interpretation of how the model differentiates between normal and abnormal heart signals. Instead of processing an entire recording as a single input, the approach divides both PCG and ECG signals into short, overlapping windows that capture local temporal dynamics. For each window, interpretable spectral and energy-based features are extracted, reflecting aspects such as band power distribution, signal amplitude, frequency centroid, and variability. These inherit the overall label of the record, creating a weakly supervised setup that allows the model to learn discriminative patterns at a finer temporal resolution. Separate XGBoost classifiers are trained for PCG and ECG windows, enabling the estimation of class probabilities corresponding to short time segments within the recording. We apply SHAP (SHapley Additive exPlanations) to trained window-level models to achieve explainability [46,47]. SHAP values quantify the contribution of each feature in a given window toward the prediction of the abnormal class. By aggregating the positive SHAP contributions along time, we get a smooth importance trajectory that aligns with the waveform for every signal. Then, these are visualized as a heatmap over the signal, highlighting regions where the model found stronger evidence of abnormal cardiac activity. This window-based visualization provides temporal interpretability without modification to the model architecture, and this gives clinicians and researchers an intuitive way to relate machine-learning decisions to physiological events in the signal. Figure 5 and Figure 6 show the obtained heatmaps for normal and abnormal classes, respectively. Figure 5 depicts the explainability analysis of normal-class ECG and PCG signals. In these visualizations, each heatmap represents the time-aligned SHAP contributions overlaid onto the original waveforms in which color intensity is used to convey the relative importance of individual time segments for the prediction of the normal class. Cooler colors (bluish) correspond to neutral or low-contribution areas, while warmer colors (yellow to red) reflect time intervals in which the model placed greater attention or confidence toward classifying a sample as normal. Across all four samples, both ECG and PCG signals show consistent periodic patterns in which heatmap activation coincides with physiologically relevant cardiac cycles. ECG heatmaps emphasize segments corresponding to QRS complexes, reflecting the model’s reliance on stable rhythmic morphology for normal class recognition. Similarly, PCG heatmaps show concentrated activation around the S1 and S2 components, suggesting that the model effectively identifies consistent timing and energy patterns indicative of healthy cardiac activity.

Figure 6 shows the explainability analysis of abnormal-class ECG and PCG signals under the same window-based SHAP interpretation framework. Heatmaps are shown that indicate how the model’s attention is dynamically shifting across time to characterize pathological segments within the signals. In contrast to the normal cases, the abnormal recordings show much sparser and more irregular activation regions, as reflected by much wider and stronger red–yellow patterns across both modalities. These highlight time intervals where the model has detected features that deviate from normal cardiac morphology or rhythm. In the case of ECG plots, the high-magnitude SHAP contributions occur at the time instants where waveforms are distorted or incoherent, reflecting the model’s sensitivity to arrhythmic behavior or waveform irregularities. The PCG heatmaps show focused activations around those segments that contain unusual intensity variations or longer systolic–diastolic intervals, typically corresponding to the presence of murmurs or turbulent flow. The simultaneous detection of such irregularities in both ECG and PCG underscores that the proposed fusion-based approach effectively integrates complementary information from electrical and acoustic domains.

### 4.3. Limitations

A limitation of this study is the lack of external validation on an independent ECG–PCG dataset. Although the proposed quality-aware fusion approach achieved promising results on the PhysioNet/CinC 2016 dataset, its generalizability to data acquired using different devices, clinical centers, patient populations, and recording protocols remains to be further investigated. Therefore, future studies should validate the proposed framework on independent external datasets.

Another limitation is that the PCG-only model already achieved strong performance on this dataset. This is expected because the PhysioNet/CinC 2016 dataset is primarily focused on heart sound classification, and PCG signals provide direct acoustic information for detecting abnormal cases. Therefore, the contribution of ECG should not be interpreted as producing a large accuracy gain under ideal PCG conditions. Instead, ECG was included as a complementary modality that may support decision making when PCG recordings are noisy, low-energy, or ambiguous. Future studies may also examine more compact feature subsets and additional feature-selection strategies to further simplify the model.

The explainability analysis should also be considered as a qualitative and supportive interpretation of the model decisions. Although the SHAP-based heatmaps highlighted physiologically meaningful ECG and PCG regions, no quantitative explainability metric or independent expert-based clinical validation was performed in this study. Therefore, these visual explanations should not be interpreted as clinically validated evidence. Future studies should include cardiologist assessment and quantitative explainability measures to confirm whether the highlighted signal regions correspond to clinically relevant cardiac abnormalities.

The proposed method also has clinical deployment-related limitations. The model was developed and tested on a single public dataset, and its performance may depend on the recording protocol, sensor type, signal quality, and patient population represented in that dataset. Therefore, the reported results should not be directly generalized to all clinical environments without further testing. Before clinical deployment, the framework should be validated prospectively on independent multi-center datasets, assessed by clinical experts, and tested under real-time acquisition conditions. In addition, integration into clinical workflow would require clear decision thresholds, uncertainty handling, and evaluation of false-positive and false-negative cases in real screening settings.

## 5. Conclusions

This study introduced a multimodal classification framework that was designed to automatically detect cardiac abnormalities by merging ECG and PCG signals through a quality-aware fusion strategy. The proposed system combines modality-specific preprocessing with spectral–temporal feature extraction and dynamic weighting based on signal reliability to ensure robust system performance even in the presence of noise. Explainability analysis further revealed that the model decisions indeed stemmed from physiologically meaningful regions, such as S1–S2 heart sound intervals and key ECG waveform components, thereby providing supportive interpretability for the model predictions. Experimental results on the PhysioNet/CinC 2016 dataset yield high diagnostic accuracy, with an average F1 score of 97.2% and accuracy of 95.6% within only 66 s for full 10-fold validation—showcasing both the precision and efficiency of the proposed approach. Future work will involve extending the fusion mechanism to accommodate more biosignal modalities and, importantly, explore adaptive attention mechanisms that adapt to different levels of noise and recording conditions. Model scalability to multi-class classification tasks, as well as real-time deployment on either embedded or wearable devices, shall be pursued. Moreover, uncertainty estimation and calibration techniques shall be incorporated to enhance reliability for clinical decision support. Finally, further explainability frameworks that combine temporal saliency with feature-level attribution shall be developed in order to provide even clearer insight into the way ECG and PCG features jointly influence cardiac abnormality detection.

## Figures and Tables

**Table 1:** Literature summary.

Authors	Method Used	Dataset Used	Performance Achieved	Limitations
Kalatehjari et al. [8]	Hybrid CNN–BiLSTM ensemble with bilinear fusion for multi-modal ECG–PCG classification.	PhysioNet/CinC Challenge 2016.	97.77% diagnostic accuracy.	High computational cost limits real-time or mobile deployment.
Chandrasekhar et al. [9]	PJM-DJRNN with intensive preprocessing and PDF-SLO feature selection.	PhysioNet/CinC 2016 (644 signals).	97.33% accuracy; F-measure ≈ 98.00%.	Focused on few disease types; broader coverage needs extra features.
Potes et al. [10]	Ensemble of AdaBoost-abstain (handcrafted features) + CNN (cycle spectro-bands).	PhysioNet/CinC 2016.	Overall score 0.8602 (winner); Sensitivity 0.9424.	Higher sensitivity at the expense of specificity.
Zabihi et al. [11]	Ensemble of 20 ANNs using features without segmentation.	PhysioNet/CinC 2016.	Overall score 0.8590 (runner-up).	Initial feature selection underweighted signal quality, hurting final score after rule change.
Chowdhury et al. [12]	5-layer DNN on MFCCs/spectrograms; DWT denoising.	PhysioNet/CinC 2016 (3240 PCG).	97.10% testing accuracy; 99.26% sensitivity.	Segmentation degrades when murmurs overlap S1/S2; needs large data.
Rubin et al. [13]	Deep CNN on MFCC heat-map images of PCG.	PhysioNet CinC 2016 training sets.	Phase II overall score 84.8%; specificity 93.1%.	Record-level decision via mean of segment probabilities is simplistic.
Tschannen et al. [14]	Wavelet-based deep features + summary stats → L2-SVM.	PhysioNet/CinC 2016 (3153 recordings).	Overall score 0.812 (hidden test).	Ternary setting with “unsure” lowered performance vs. binary.
González Ortiz et al. [15]	MFCC + Intra-DTW temporal features → linear SVM.	PhysioNet/CinC 2016.	82.4% on hidden test.	Linear SVM may underfit; non-linear models could help.
Homsi et al. [16]	Nested ensemble: Random Forest + LogitBoost + cost-sensitive classifier over 131 features.	PhysioNet/CinC 2016.	84.48% overall (5th).	Strongly dependent on recording quality; class imbalance limits minority learning.
Nilanon et al. [17]	CNN on overlapping 5 s segments (spectrograms/MFCCs) with voting.	PhysioNet/CinC 2016.	Overall score 0.811; sensitivity 77.0%.	Low sensitivity; few poor-quality samples hinder “unsure” detection.
Chakir et al. [18]	SVM on features from synchronized ECG + PCG vs. PCG-only.	CinC 2016 Dataset A (100 recs).	92.5% accuracy; AUC 0.9505 (fusion).	Small sample and single subset limit generalizability.
Vernekar et al. [19]	Markov-chain transition features + XGBoost/ANN ensemble.	PhysioNet 2016.	81.75% (validation).	Nonstationary noise not fully removable in frequency domain.
Grzegorczyk et al. [20]	Neural networks over 48 time/ordinate/frequency features with quality gating.	PhysioNet 2016 training set.	Final score 0.79 (sens 81%, spec 76%).	Lack of stethoscope location metadata limits pathology-specific tuning.
Goda et al. [21]	SVM using morphological + frequency + wavelet-envelope features after segmentation.	PhysioNet/CinC 2016.	80.28% MAcc (entire test).	Needs stronger noise immunity and robustness to varied recording conditions.

**Table 2:** Performance evaluation of the proposed model based on F1-score, ACC, Spec, and Sens.

Fold	Model	F1-Score (%)	ACC (%)	Spec (%)	Sens (%)
1	ECG	90.5	84.2	40.2	97.4
	PCG	97.6	96.3	91.5	97.8
	FUSE	98.4	97.5	92.7	98.9
2	ECG	88.7	81.1	30.5	96.3
	PCG	95.9	93.5	78.0	98.2
	FUSE	96.6	94.6	84.1	97.8
3	ECG	89.7	82.8	32.9	97.8
	PCG	96.7	94.9	85.4	97.8
	FUSE	97.3	95.8	87.8	98.2
4	ECG	88.7	81.1	30.5	96.3
	PCG	97.0	95.5	92.7	96.3
	FUSE	97.2	95.8	93.9	96.3
5	ECG	91.1	85.3	45.1	97.4
	PCG	96.9	95.2	86.6	97.8
	FUSE	98.0	96.9	93.9	97.8
6	ECG	90.4	83.9	34.1	98.9
	PCG	98.4	97.5	91.5	99.3
	FUSE	98.7	98.0	95.1	98.9
7	ECG	89.0	81.6	32.1	96.3
	PCG	96.1	94.1	87.7	96.0
	FUSE	96.2	94.1	86.4	96.3
8	ECG	89.9	83.1	33.3	97.8
	PCG	96.1	93.8	75.3	99.3
	FUSE	96.0	93.8	81.5	97.4
9	ECG	88.1	79.9	24.7	96.3
	PCG	96.6	94.6	85.2	97.4
	FUSE	96.7	94.9	86.4	97.4
10	ECG	90.2	83.6	37.0	97.4
	PCG	96.4	94.4	86.4	96.7
	FUSE	96.8	94.9	84.0	98.2

**Table 3:** Average evaluation metrics.

Model	Metric	Average (Avg) ± Std Dev (%)	Final OOF (%)
**ECG**	F1-Score	89.6 ± 0.9	89.7
	ACC	82.7 ± 1.6	82.7
	Spec	34.1 ± 5.4	32.6
	Sens	97.2 ± 0.8	97.8
**PCG**	F1-Score	96.8 ± 0.7	96.8
	ACC	95.0 ± 1.2	95.0
	Spec	86.0 ± 5.3	86.0
	Sens	97.7 ± 1.1	97.7
**FUSE (Quality-Aware)**	F1-Score	97.2 ± 0.9	97.1
	ACC	95.6 ± 1.4	95.6
	Spec	88.6 ± 4.7	88.5
	Sens	97.7 ± 0.8	97.7

**Table 4:** The obtained performance evaluation scores for ablation studies.

	F1-Score (%)	ACC (%)	SPEC (%)	SENS (%)
PCG only (MFCC on)	96.80	95.00	86.00	97.70
ECG only + Guard	80.50	76.60	42.80	86.70
ECG only (no Guard)	80.50	76.60	42.80	86.70
ECG only (no wavelet)	80.50	76.60	42.80	86.70
PCG only (no MFCC)	95.40	92.80	81.90	96.10
ECG only (Standard Scaler)	80.50	76.60	42.80	86.70
Fusion (quality-aware + Guard)	96.80	95.10	85.40	97.90
Fusion (simple avg no Guard)	97.20	95.70	88.20	97.90
Fusion + Calibration	97.30	95.80	87.90	98.20

**Table 5:** Comparative analysis of the proposed method with the literature papers on the same dataset.

Authors	Year	Modality	Method	ACC (%)
Cheng et al. [39]	2021	ECG	A combination of a 24-layer DCNN and BiLSTM	89.3
Chowdhury et al. [12]	2018	PCG	A 5-layer DNN	97.1
Li et al. [40]	2022	ECG + PCG	BiLSTM-CNN	96.1
Chakir et al. [18]	2020	ECG + PCG	Machine learning methods	92.5
Singh et al. [41]	2021	ECG + PCG	SVM, KNN and Ensemble methods	93.1
Schölzel et al. [45]	2016	ECG + PCG	LSTM	82.3
Chandrasekhar et al. [9]	2025	ECG + PCG	PJM-DJRNN	97.3
Proposed	2025	ECG + PCG	Quality-Aware Fusion	95.6

## Data Availability

Data are publicly available at https://physionet.org/content/challenge-2016/1.0.0/ (accessed on 17 July 2025).

## Code Availability

The source code supporting the findings of this study may be obtained from the corresponding author upon reasonable request.
